# Detecting Critical Damage in Concrete by Taking Advantage of Acoustic Events with an Amplitude Exceeding Their Mean Value

**DOI:** 10.3390/ma19061264

**Published:** 2026-03-23

**Authors:** Dimos Triantis, Ilias Stavrakas, Ermioni D. Pasiou, Stavros K. Kourkoulis

**Affiliations:** 1 Electronic Devices and Materials Laboratory, Faculty of Engineering, Department of Electrical and Electronics Engineering, Ancient Olive Grove Campus, University of West Attica, 250 Thivon Avenue, Egaleo, 122 44 Athens, Greece; triantis@uniwa.gr (D.T.); ilias@uniwa.gr (I.S.); 2 Laboratory for Testing and Materials, Department of Mechanics, School of Applied Mathematical and Physical Sciences, Zografou Campus, National Technical University of Athens, 157 73 Athens, Greece; epasiou@central.ntua.gr

**Keywords:** acoustic emissions, acoustic events, natural time, concrete, fiber-reinforced concrete, damage

## Abstract

**Highlights:**

- A time series of acoustic events, the amplitude of which exceeds the respective average value, is studied.
- The evolution of their cumulative number is considered versus natural time.
- The slope of this function attains a constant value slightly before the applied load attains its maximum value.
- Attainment of this constant value suggests that acoustic events are now produced with increased energy content.
- From this instant on, the networks of microcracks become richer, leading inevitably to their mutual coalescence.
- Thus, attainment of the above constant value signals entrance to the critical stage of impending fracture.

**Abstract:**

A novel approach for detecting preliminary signals designating upcoming entrance of a loaded system to the critical stage of impending fracture is assessed. The approach is based on the analysis of a time series of the cumulative number of acoustic events, the amplitude of which exceeds the respective average value of all the events recorded during loading. Using the “sliding window” technique, the average slope of the evolution of this time series is quantified, either against conventional or natural time (the latter provides a more detailed view of the stage before macroscopic fracture, during which the “information” gathered is very densely packed in a short interval). For the needs of this study, data from a previously published experimental protocol are exploited. The protocol comprised notched, beam-shaped specimens, made of either plain or fiber-reinforced concrete, under three-point bending. It is concluded that the slope of the evolution of the above time series systematically attains a value equal to unity slightly before the applied load attains its peak value. The results of the present analysis are in qualitative agreement with the respective ones based on either the instantaneous frequency of generation of acoustic events or the Euclidean distance between the sources of acoustic signals.

## 1. Introduction

The Acoustic Emissions (AEs) technique is the most mature and widely used structural health monitoring tool worldwide. Its practical application, either for laboratory experiments or for structural applications (structural health monitoring), was long ago standardized. Especially for concrete structures, one could mention the respective standards by ASTM [1] and the respective recommendations by RILEM [2]. In addition, one could mention the well-cited volume by Grosse et al. [3]. Among the main advantages of the AEs technique is the fact that it provides information from the interior of a loaded structural element [3,4,5]. These stress redistributions are caused by sudden changes in the internal structure of the material due, for example, to the generation of microcracks or their coalescence in the direction of forming fatal macrocracks. This inherent correlation between the signals emitted and the degradation of the mechanical properties of the materials (due to the development of the networks of microcracks) provides information regarding the degree (level) of internal damage and the closeness of the loaded element to the critical stage of impending macrocrack propagation [6,7,8,9].

The need for early warning signals about the upcoming entrance to the stage of impending fracture is imperative for structures made of cementitious materials, especially concrete, due to the increased brittleness of such materials, rendering the phenomenon of fracture abrupt and catastrophic. These materials are broadly used in structural engineering applications due to their increased compressive strength, their low cost, and the fact that structures of complex shapes can be prepared relatively easily [10]. Their fracture is nowadays confronted as a complex dynamic process,. which is closely related to the onset of development of internal networks of microcracks and their subsequent mutual coalescence [11].

A large number of relatively recent scientific studies have highlighted that the gradually increasing number of microcracks, which are generated as the applied load increases beyond the linearity level (as well as their gradually increasing length), is interrelated to an increased number of acoustic sources, or, equivalently, to the increased rate of generation of acoustic events with increased amplitude and energy content, and, finally, to an increased number of the respective counts. Especially during the last loading stage (i.e., as the applied load approaches its peak value), it has been indicated that the rate of generation of acoustic events exhibits a strong increasing trend, which is considered as a signal for the upcoming generation of a fatal macrocrack and the final disintegration of the specimen [12,13,14,15]. Along the same lines, a rapid increase in the rate of the counts recorded [12,16,17], or of the energy content of the acoustic signals [18,19,20,21] are sometimes considered alternative warning signals about upcoming macroscopic fracture.

It is interesting to note at this point that the signal strength distribution of the acoustic emissions exhibits statistical characteristics similar to those exhibited by the magnitude of seismic excitations (see, for example, refs. [22,23,24]). In this context, the familiar b-value analysis according to Gutenberg and Richter [25] is successfully applied, also, for the analysis of the acoustic signals [26,27,28]. Concerning structures (or specimens) made of concrete, it is proven that while their load carrying capacity tends to be exhausted, the b-values exhibit a systematic decrease towards a value equal to unity, a response that is attributed to the fast and/or unstable development of microcracks, since the acoustic activity appears to be intensified and acoustic signals of increased amplitude are recorded [26,29,30].

Within the framework of the above discussion, the present study aims to assess a new method for detecting signals heralding imminent fracture. This method is based on the study of the evolution of the time series of the cumulative number of acoustic events, the amplitude of which exceeds the respective average value of all the events recorded during the whole loading procedure. The evolution of this time series is depicted both in the conventional time domain and also in that of natural time. The concept of natural time was introduced by Varotsos et al. [31,32,33] in their effort to describe the conditions under which a system enters a new state during which an extreme event is about to appear. It is nowadays accepted that natural time is a flexible tool for the analysis of the response of complex systems in a broad variety of disciplines, ranging from mechanics and physics to medicine and finance [34]. The relative literature is very rich, especially in the discipline of mechanics of materials. Quite a few studies are published, which reveal that for mechanically loaded systems, the analysis of the acoustic signals in terms of the natural time concept permits the safe detection of critical stages, signaling early for upcoming fracture [8,34,35,36,37,38,39]. Quite a few parameters of acoustic activity, like the average rate of generation of events [15] and the cumulative number of counts [17], have been analyzed in the natural time domain and it is systematically proven that the specific analysis provides clearly distinguishable pre-failure indicators.

In the present study, advantage was taken of data from an already published experimental protocol [40,41] with notched, beam-shaped specimens, made of either plain or fiber-reinforced concrete, under three-point bending. It is proven that the slope of the evolution of the time series of events of amplitude higher than the respective mean value of all the events recorded attains systematically a value equal to unity as the applied load tends to be maximized, providing a potentially interesting pre-failure index. The novelty of the study is that it highlights the fact that the average slope of the above time series is independent of the rate of the generation of events and that it depends exclusively on the rate of generation of events of high amplitude. The conclusions that will be derived aim to support campaigns on concrete and fiber-reinforced concrete specimens at laboratory scale. Any potential extension to specimens made of other materials, or to specimens subjected to different loading schemes, or at larger scales, should be exercised with the utmost caution—or, preferably, only after the respective database has been sufficiently expanded with data from additional experimental protocols.

The manuscript is structured formally. Initially, the materials tested and the experimental protocol are shortly described. The analysis of the methodology introduced for the detection of pre-failure indices follows. Then, the experimental results are discussed thoroughly within the frame of the approach introduced here. Critical discussion of the outcomes of the study follows, together with a comparative assessment of them versus the respective ones of traditional approaches. Finally, the main conclusions drawn are comprehensively described.

## 2. Materials and Methods

### 2.1. The Material, the Specimens and the Experimental Protocol

The data used in the present study refer to the AEs that were recorded during an experimental protocol with centrally pre-notched specimens, made of either plain or fiber-reinforced concrete subjected to three-point bending [40,41]. The indicative composition of the concrete used for the preparation of the specimens is shown in Table 1. Concerning the curing procedure, the requirements of the EN 12390-2 standard [42] were adopted, and the specimens after demolding were immersed in water at a constant temperature of about 18 °C.

All specimens were of prismatic shape, and they were mechanically notched at their central section. A detailed description of the geometry and composition of the specimens can be found in previously published papers by the authors’ team, in which the spatiotemporal distribution of the acoustic sources [40] and the correlation between acoustic and electrical signals [41] were studied. The acoustic activity was detected and recorded with the aid of eight acoustic sensors of the R15a type and preamplifiers with 40 dB gain were used. The acquisition system used was the PCI-2, while the software used was the AEWin. Both the hardware and the software used were by Mistras Group, Inc., Princeton Junction, NJ, USA.

**Figure 1:**
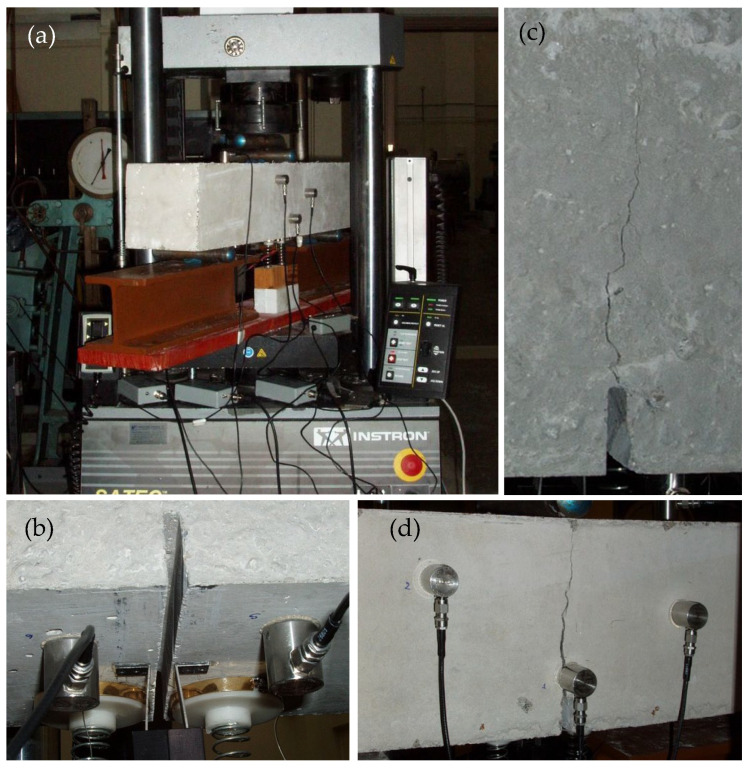
(**a**) An overview of the experimental set-up with a typical specimen just before loading; (**b**) the lower side of a typical specimen, together with the acoustic sensors and the clip-gauge used to measure the Notch Mouth Opening Displacement (NMOD); (**c**,**d**) the front face of typical fiber-reinforced specimens at various stages of the slow crack-front propagation motif [41].

The specimens tested were classified into four groups, depending on their composition. The first group included specimens made of plain concrete (denoted from here on as C class). The remaining three groups included specimens reinforced with short fibers made either of steel (CM class), or of polyolefin (CP/F class) or of polypropylene (CP/P class). The length and diameter of the steel fibers were equal to about 50 mm and 1.30 mm, respectively, while their tensile strength was estimated to about 690 MPa. The respective figures for the polyolefin fibers were 60 mm, 0.84 mm, 465 MPa, and for the polypropylene ones 50 mm, 0.75 mm and 620 MPa. Further details about the characteristics of the reinforcing fibers can be found in refs. [40,43,44,45]. Some characteristic photos of the experimental set-up and fractured specimens are shown in Figure 1.

**Figure 2:**
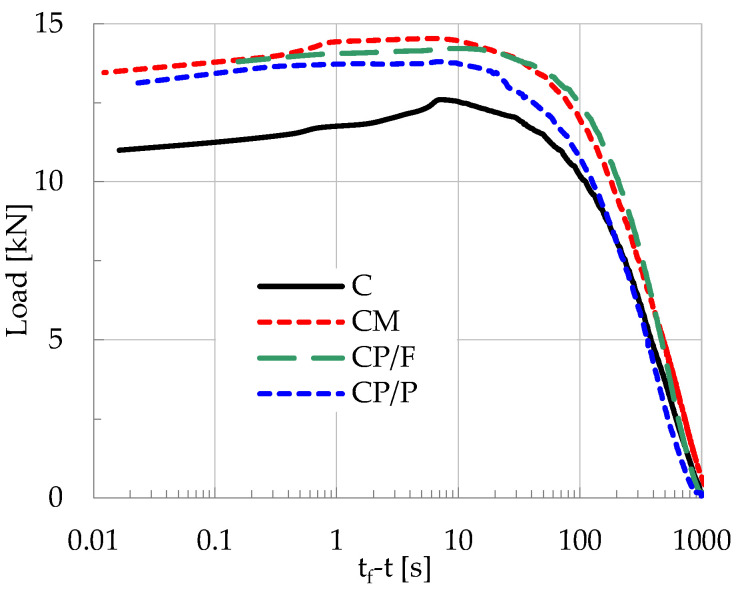
The evolution of the applied load, for typical specimens of each class, versus the time-to-failure parameter, t_f_-t, along logarithmic scale.

Four specimens per class were tested with the aid of a very stiff Instron-Satec servo-hydraulic loading frame with a capacity of 300 kN (Norwood, MA, USA). Displacement-controlled conditions were induced at a constant rate equal to 0.08 mm/min. The temporal evolution of the applied load, for typical specimens of each class, is shown in Figure 2 versus the time-to-failure parameter, t_f_-t (along logarithmic scale), where t_f_ denotes the instant of fracture. Using the specific time parameter permits magnified insight into what happens during the very last stage before the fracture of the specimens, which is the most critical for the needs of the present study. It is important to note that the instant, t_Lmax_, at which the applied load is maximized does not coincide with the instant of fracture (t_f_) of the specimens, but rather it precedes it by approximately 6 to 10 s, depending on the specific experiment.

**Figure 3:**
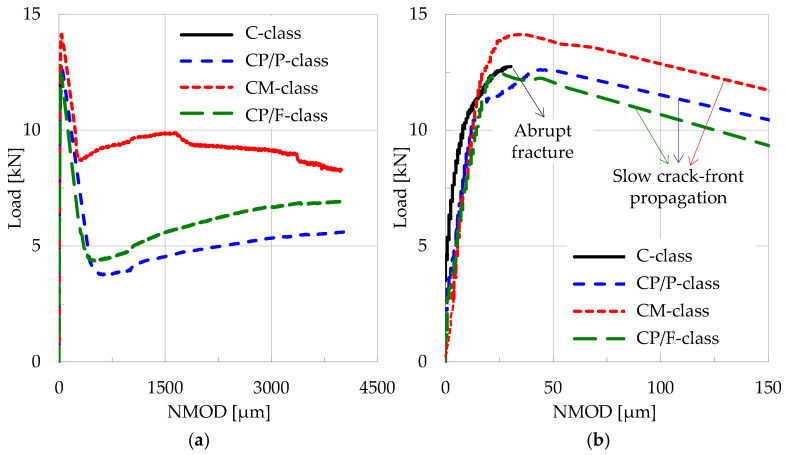
(**a**) The evolution of the applied load, for typical specimens of each class, versus the Notch Mouth Opening Displacement (NMOD); (**b**) magnified view for small values of the NMOD, highlighting the critical differences between the plain concrete specimens (abrupt fragmentation into two pieces) and the fiber-reinforced ones (slow propagation of the macroscopic crack front).

In order to exhibit the response of each class of materials to the loading scheme of three-point bending, the applied load is plotted in Figure 3 in juxtaposition to the Notch Mouth Opening Displacement (NMOD), for a characteristic specimen from each class. 

**Figure 4:**
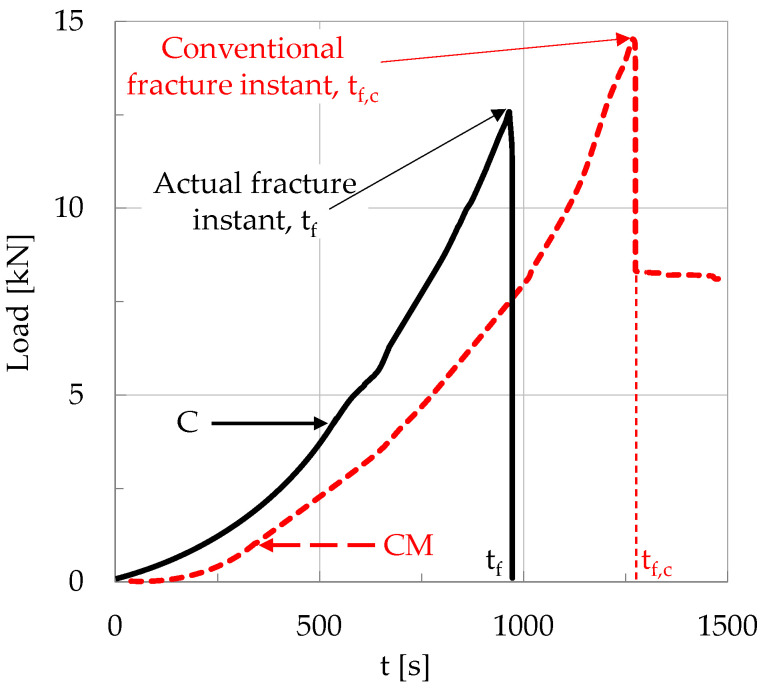
The difference between the actual fracture instant, t_f_, of specimens made of plain concrete and the “conventional” fracture instant, t_f,c_, of specimens made of fiber-reinforced concrete.

It is clearly seen from this figure (and also from Figure 1) that there is a crucial difference between the experiments with plain concrete specimens (which were suddenly fractured into two pieces without any exception) and the experiments with fiber-reinforced specimens, for which slow propagation of a macroscopic crack front was observed, starting from the crown of the notch and directed towards the load application point. In this context, the term “fracture instant” is not literally correct for the fiber-reinforced specimens. Indeed, for these specimens there is not an instant at which they suddenly disintegrate into two or more parts. What actually happens is that at a given instant, a macrocrack starts propagating relatively slowly (originating from the crown of the notch) towards the point of load application. At this time instant, the applied load exhibits an abrupt drop, but it does not zero suddenly (as it happens for the specimens made of plain concrete), and the two “fragments” are kept together due to the action of the reinforcing fibers. In this context, from this point on, the instant of the abrupt load drop is considered as the fracture instant for the reinforced specimens. The difference between the actual fracture instant, t_f_, of the plain concrete specimens and the “conventional” fracture instant, t_f,c_, of the reinforced ones is shown in Figure 4.

### 2.2. Synoptic Outline of the Concept of Natural Time

The concept of natural time was initially introduced in the field of seismology and earthquake engineering [31] in an effort to describe the response of complex, dynamic systems while they were about to enter the stage of generation of extreme disastrous events. Gradually it became a flexible and reliable tool, widely used for the description of the dynamic evolution of such systems since it reduces uncertainty and allows optimal extraction of signal information, especially from systems approaching criticality [33,34].

The key idea behind the analysis in terms of natural time is that only the sequence (order) of the elements of the series (either acoustic events or earthquakes) must be taken into account (together with their energy content). The reason is that attention is focused on the changes induced to the complex system due to the external loading, which is not part of the system. In this context, the analysis in the natural time domain retains only the order of the events and not the conventional time they occurred. According to the underlying theoretical principles of the analysis by means of the natural time concept, in a time series of N events the natural time serves as the index for the occurrence of the k^th^ event. Thus, the first event is ‘placed’ at χ_1_ = 1/N, the second at χ_2_ = 2/N and so on, until the last (N^th^) one which is placed at χ_N_ = N/N = 1. Then, each event can be paired to a characteristic quantity Q of the elements of the time series.

**Figure 5:**
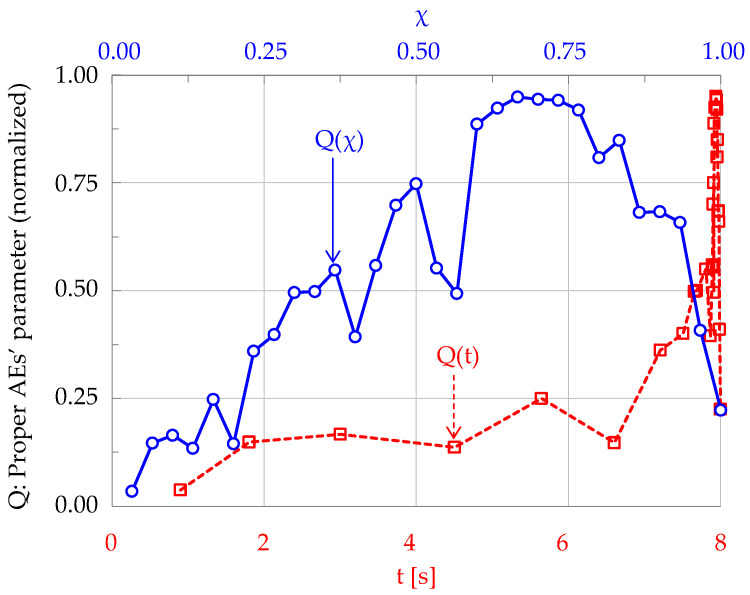
The evolution of a normalized (over its maximum value) parameter Q in the conventional (red dashed line) and natural time domains (blue continuous line).

In order to make this procedure clear, consider a time series including N = 30 acoustic events, recorded from the onset of a given experiment (t = 0) up to the instant of fracture (t = 8.0 s). Each event is paired to the respective characteristic quantity Q_i_, the evolution Q(t) of which in terms of conventional time t is plotted in Figure 5 (red dashed line).

As expected, the vast majority of events are recorded during the very last loading stage before fracture, and therefore the information is here densely packed in an extremely narrow time interval, the width of which is usually a very small portion of the overall duration of the test, rendering its exploration difficult. On the other hand, plotting the same time series versus the natural time χ, one obtains a uniform distribution Q(χ) of the elements (blue, continuous line in Figure 5) of the series over the range of χ-values (0 < χ ≤ 1), permitting optimum exploitation of the information provided by the specific time series.

### 2.3. Methodology

Assume that up to the instant t_i_ (when the ith acoustic event was recorded), the number of events with amplitude exceeding the average $\bar{A}$ of the amplitudes of all the events recorded until the fracture instant t_f_ (actual or conventional) is equal to $CN_{A\geq \bar{A}}$. This number is normalized over its maximum value (i.e., the number of events with amplitude exceeding $\bar{A}$ that are recorded until the instant t_f_), and is denoted as $CN_{A\geq \bar{A}}^{*}$.

In order to “transfer” the time series of $CN_{A\geq \bar{A}}$ (or that of $CN_{A\geq \bar{A}}^{*}$) from the conventional time domain to the natural time one, assume that during a given experiment the time series of the amplitudes of the N acoustic events recorded in total is:

(1) $$A\left(t_{1}\right),\;A\left(t_{2}\right),\;\ldots ,\;A\left(t_{k}\right),\;\ldots ,\;A\left(t_{N-1}\right),\;A\left(t_{N}=t_{f}\right).$$

In natural time the above time series becomes:

(2) $$A\left(\chi_{1}\right),\;A\left(\chi_{2}\right),\;\ldots ,\;A\left(\chi_{k}\right),\;\ldots ,\;A\left(\chi_{N-1}\right),\;A\left(\chi_{N}\right),$$

where $\chi_{1}=\frac{1}{N},\;\chi_{2}=\frac{2}{N},\;\ldots ,\;\chi_{k}=\frac{k}{N},\;\ldots ,\;\chi_{N-1}=\frac{N-1}{N},\;\ldots ,\;\chi_{N}=\frac{N}{N}\;=\;1$. Thus, the time series of the cumulative acoustic events in natural time is written as:

(3) $$\mathrm{CN}\left(\chi_{1}\right)\;=\;1,\;\mathrm{CN}\left(\chi_{2}\right)\;=\;2,\;\ldots ,\;\mathrm{CN}\left(\chi_{k}\right)\;=\;k,\;\ldots ,\;\mathrm{CN}\left(\chi_{N-1}\right)\;=\;N-1,\;\mathrm{CN}\left(\chi_{N}\right)\;=\;N,$$

and that of the cumulative acoustic events of amplitude exceeding $\bar{A}$ is written as:

(4) $$CN_{A\geq \bar{A}}\left(\chi_{1}\right),\;CN_{A\geq \bar{A}}\left(\chi_{2}\right)\;,\ldots ,\;CN_{A\geq \bar{A}}\left(\chi_{k}\right),\;\ldots ,\;CN_{A\geq \bar{A}}\left(\chi_{N-1}\right),\;CN_{A\geq \bar{A}}\left(\chi_{N}\right)\;=\;n,$$

where n is the total number of acoustic events with $A\;\geq \bar{\;A}$, recorded until the termination of the experiment (i.e., the fracture of the specimen, or, equivalently, the instant t_f_). It is to be noted here that in case of the amplitude of the kth event holds that $A\left(\chi_{k}\right)\;<\;\bar{A},$ then $CN_{A\geq \bar{A}}\left(\chi_{k}\right)=CN_{A\geq \bar{A}}\left(\chi_{k-1}\right)$. Finally, the time series of the normalized (over the maximum value n) cumulative acoustic events with amplitude exceeding $\bar{A}$ becomes:

(5) $$CN_{A\geq \bar{A}}^{*}\left(\chi_{1}\right),\;CN_{A\geq \bar{A}}^{*}\left(\chi_{2}\right),\;\ldots ,\;CN_{A\geq \bar{A}}^{*}\left(\chi_{k}\right),\;\ldots ,\;CN_{A\geq \bar{A}}^{*}\left(\chi_{N-1}\right),\;CN_{A\geq \bar{A}}^{*}\left(\chi_{N}\right)\;=\;n.$$

While plotting the evolution of either $CN_{A\geq \bar{A}}$ or $CN_{A\geq \bar{A}}^{*}$ in terms of conventional time, the “distance” (time interval) between any two successive values is equal to the interevent time of these two values (which is a non-constant quantity). On the contrary, plotting the evolution in terms of natural time, all values of either $CN_{A\geq \bar{A}}$ or $CN_{A\geq \bar{A}}^{*}$ are equidistant, and the distance between any two of them is equal to Δχ = 1/n.

Taking now into account that during a given experiment the values of $CN_{A\geq \bar{A}}^{*}$ increase monotonically from 0 to 1, the present study is focused on the analysis of the variation in the average rate of change (denoted from here on as ${\bar{m}}_{A}$) of $CN_{A\geq \bar{A}}^{*}$ rather than to the changes of $CN_{A\geq \bar{A}}^{*}$ itself. The principal advantage of this approach is that the values of ${\bar{m}}_{A}$ are independent of the rate of generation of acoustic events depending only on the average number of events, for which it holds that $A\;\geq \bar{\;A}$. In addition, the fact that the values of the elements of the $CN_{A\geq \bar{A}}^{*}$ time series in the natural time domain are equidistant during all stages of the loading procedure offers a chance for “thinner” resolution of the changes of ${\bar{m}}_{A}$ during the various stages of loading, especially during the last instants before fracture, which are characterized by increased rates of production of acoustic events (with high energy content).

The values of ${\bar{m}}_{A}$ are determined as the slope (versus χ) of the linear trendline of a constant number of k successive $CN_{A\geq \bar{A}}^{*}$ values. Then, sliding the “window” of the k values by a “sliding step” of Δk events (Δk may be equal to either one or more events, depending on the specific time series studied), one determines the next value of ${\bar{m}}_{A}$ and so on. Each ${\bar{m}}_{A}$ value is then paired to either the average value $\bar{t}$ of the time instants at which the k events of the specific “window” were recorded or the respective mean value $\bar{\chi }$ of the natural times, χ_i_, of the elements building the specific “window” (group of events). Adopting a similar procedure, one determines the respective mean value of the applied load corresponding to each “window” (or, equivalently, to each ${\bar{m}}_{A}$ value), which is paired again either to $\bar{t}$ or to $\bar{\chi }$.

## 3. Results

The discrepancies between specimens of the same class were relatively small, concerning both the mechanical and the acoustic data. Numerical values of characteristic quantities are recapitulated in Table 2, which includes: the maximum load attained (L_max_), the time interval between the instants of load maximization and fracture (t_f_-t_Lmax_), the overall number of acoustic events recorded (N), the average value of the amplitude of all the N acoustic events ($\bar{A}$), the number, n, of acoustic events the amplitude A of which fulfills the condition $A\;\geq \;\bar{A}$, and finally, the portion of n with respect to N. In addition (and for convenience reasons), typical characteristics of the reinforcing fibers (namely, their type, content, length, diameter and tensile strength) are also recorded in Table 2.

**Figure 6:**
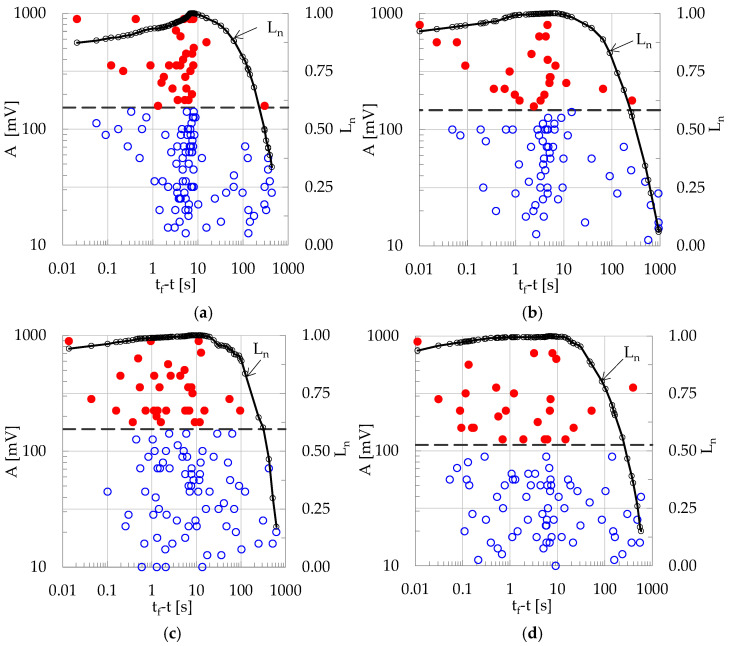
The amplitudes of the events versus the time-to-failure (t_f_-t), along logarithmic scales, in juxtaposition to the evolution of the respective normalized load, L_n_. (**a**) C class; (**b**) CM class; (**c**) CP/F class and (**d**) CP/P class. Empty (blue) symbols denote events with $A<\bar{A}$, and filled (red) symbols events with $A\geq \bar{A}$. The black dashed line indicates the average amplitude for all the N events.

The temporal evolution of the amplitudes of all the N acoustic events (recorded during the overall loading procedure) for one characteristic experiment from each class is plotted in Figure 6, versus the time-to-failure (t_f_-t), along logarithmic scales. The respective evolution of the normalized load L_n_ is also plotted. The average value of the amplitudes of all the events is denoted by a dashed (black) horizontal line. The events of amplitude below the respective average value are denoted by empty (blue) symbols, while the ones with amplitude exceeding the average value are denoted by filled (red) symbols.

**Figure 7:**
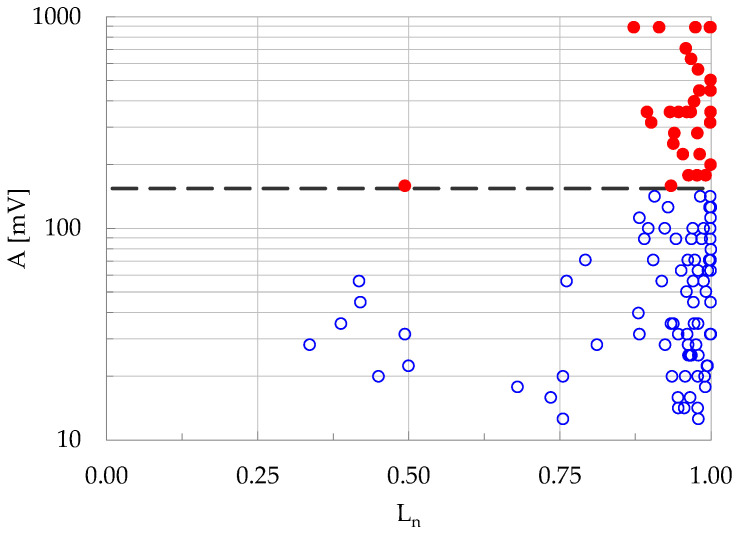
The amplitudes of the events versus the applied load (normalized over its peak value) for a characteristic specimen made of plain concrete (C-class). Empty (blue) symbols denote events with $A\;<\;\bar{A}$ and filled (red) ones denote events with $A\;\geq \;\bar{A}$. As in Figure 6, the black dashed line indicates the average amplitude for all the N events recorded during the specific experiment.

As expected, during the early loading stages the events with $A\geq \bar{A}$ are quite rare. Especially for the plain concrete specimen (C class), it is mentioned characteristically that only one acoustic event with amplitude exceeding the respective average value was recorded until the instant t_f_-t ≈ 15 s, i.e., until about fifteen seconds before the fracture of the specific specimen. It is only during the last eight seconds that a significant number of events with $A\geq \bar{A}$ are recorded. The above conclusion is further supported by Figure 7, in which the amplitudes of all the events recorded is plotted versus the (normalized) applied load L_n_ for a typical specimen made of plain concrete (C class). It is obvious that during the early loading stages the specimen is either totally silent or the acoustic activity is extremely weak. Acoustic events with amplitude exceeding the average value $\bar{A}$ are recorded after the applied load exceeds about 87% of its peak value. The overall picture is quite similar, also, for all the fiber-reinforced specimens without any exception, independently of the nature of the reinforcing fibers.

In the next subsections, the results of the above four experiments will be analyzed in depth. The analysis for the plain concrete class will be somewhat more extensive, in order for the procedure introduced in the present study to become clear. For the remaining three classes, the analysis will be focused mainly on the evolution of the parameter ${\bar{m}}_{A}$ during the whole procedure, i.e., from the onset of loading up to the fracture of the specimens.

### 3.1. The Evolution of the Parameter ${\bar{m}}_{A}$ for the Plain Concrete Specimens

**Figure 8:**
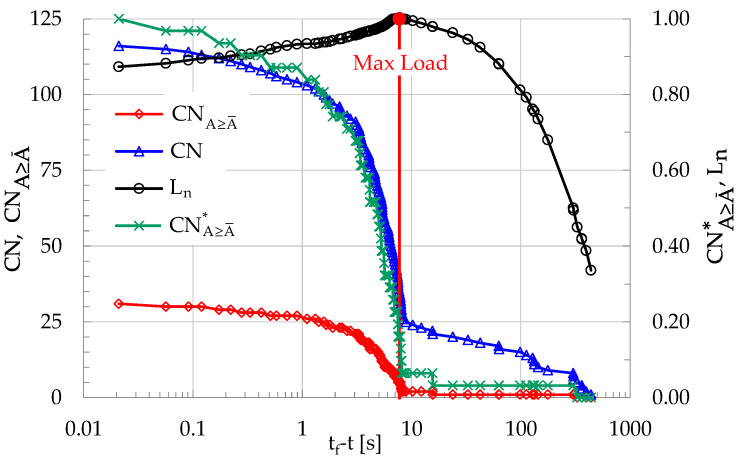
The evolution of the cumulative number of events CN, and that of the cumulative number of events $CN_{A\geq \bar{A}}$ with $A\geq \bar{A}$ versus t_f_-t. The variation in the normalized version $CN_{A\geq \bar{A}}^{*}$ of the latter is plotted (secondary vertical axis), together with the respective one of the normalized load.

During the specific experiment discussed here, n = 31 acoustic events with $A\;\geq \;\bar{A}$ were recorded, corresponding to about 27% of the overall number N of events, which was equal to N = 116. The cumulative number of events CN with amplitude exceeding the threshold of 10 mV (40 dB) are plotted in Figure 8 versus the time-to-failure (t_f_-t) parameter, along a logarithmic scale. In the same figure the respective evolution of the cumulative number of events with $A\;\geq \bar{\;A},$ denoted as $CN_{A\geq \bar{A}}$, is also plotted together with its normalized version $CN_{A\geq \bar{A}}^{*}$ along the secondary vertical axis. Along the same axis (i.e., the secondary vertical axis) the normalized load L_n_ is also plotted.

**Figure 9:**
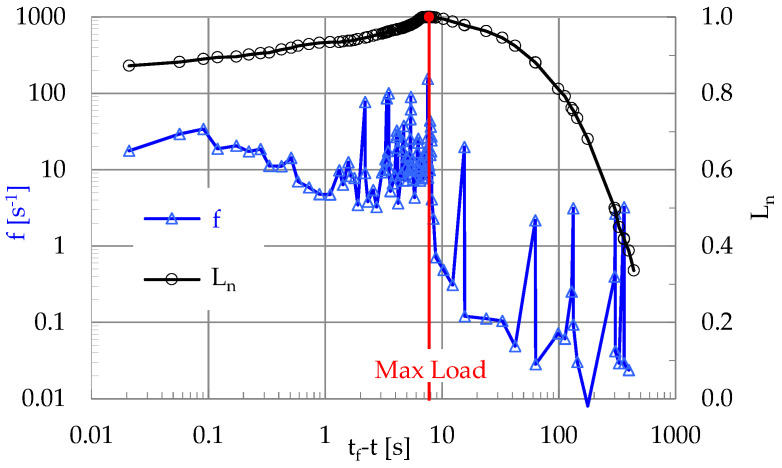
The evolution of the instantaneous frequency of generation of acoustic events versus the logarithm of the time-to-failure, in juxtaposition to the respective evolution of the applied load.

The most striking feature that characterizes the evolution of the quantities plotted in Figure 8 is that the cumulative number of events with $A\;\geq \bar{\;A}$ exhibits an abrupt increase as the load tends to its peak value, which is seen vividly considering the evolution of its normalized value $CN_{A\geq \bar{A}}^{*}$. This abrupt increase of $CN_{A\geq \bar{A}}^{*}$ (or, equivalently of the non-normalized $CN_{A\geq \bar{A}}$) is attributed to the increased rate of generation of events as the applied load tends to be maximized, or, in other words, as the fracture instant approaches. The above conclusion is further supported by the temporal evolution of the instantaneous frequency, f, of generation of acoustic events, which is defined as the inverse of the interevent time interval, δτ, between any two events, i.e., f = 1/δτ. For the experiment discussed here, the evolution of f is plotted in Figure 9, versus the logarithm of t_f_-t.

It is seen from Figure 9 that, as long as the applied load is lower than its respective peak value, the instantaneous frequency, f, attains values lower (as an average) than unity (with some strong fluctuations). However, as the load tends to attain its peak value, f exhibits a sudden increase, ranging now around f ≈ 10 s^−1^, with a stabilizing trend.

**Figure 10:**
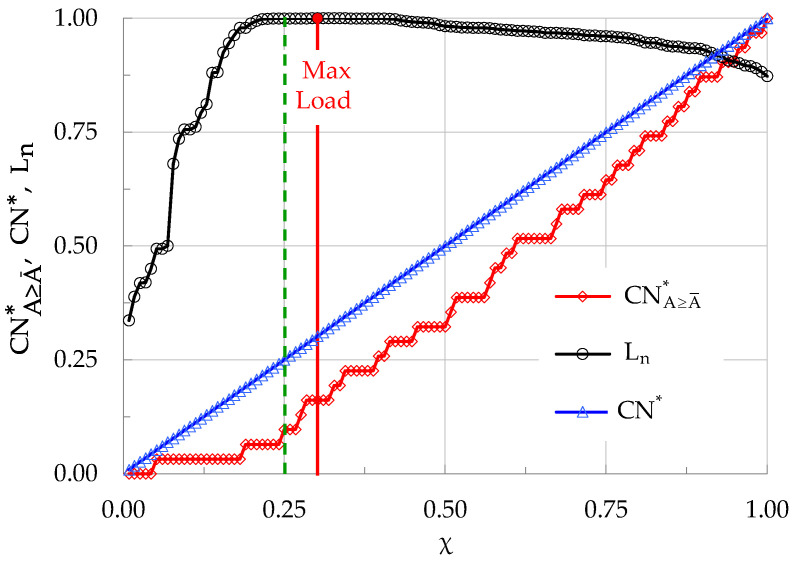
The evolution of $CN_{A\geq \bar{A}}^{*}$ in the natural time domain (i.e., versus the dimensionless parameter χ), together with the respective evolution of CN* and L_n_, for a plain concrete specimen.

As a next step, the evolution of $CN_{A\geq \bar{A}}^{*}$ is studied in the natural time domain, i.e., versus the parameter χ, as is shown in Figure 10. In the same figure, the evolution of normalized cumulative events, CN*, and that of the normalized load, L_n_, are also shown. As expected, in the natural time domain the evolution of CN* (i.e., in terms of χ) is just a straight line with a slope equal to 1 (in fact, it is the bisector of the plot). It will be proven in the next sections that the specific value of the constant slope of CN* is an index which distinguishes the intervals with increased generation of events $A\;\geq \bar{\;A}$ (slope exceeding one) from intervals with increased generation of events with $A\;<\bar{\;A}$ (slope smaller than unity). Concerning $CN_{A\geq \bar{A}}^{*}$, its evolution is now much smoother compared to that observed in Figure 8, i.e., in the conventional time domain. As already mentioned, the values of $CN_{A\geq \bar{A}}^{*}$ in the natural time domain do not depend on the rate of generation of acoustic signals in general, but rather they depend exclusively on the rate of generation of events with $A\geq \bar{A}$. Comparing Figure 10 with Figure 8, it can be seen that in the conventional time domain the abrupt increase of $CN_{A\geq \bar{A}}^{*}$ is observed at the instant t_f_-t ≈ 8 s (which is, in fact, the instant of load maximization). In the natural time domain, the respective instant at which a clearly noticeable increase of $CN_{A\geq \bar{A}}^{*}$ is observed is equal to χ ≈ 0.25 (see the dashed green vertical line in Figure 10). This natural time instant precedes the instant of load maximization, which for the specific specimen was observed at about χ ≈ 0.30 (see the continuous red vertical line in Figure 10).

**Figure 11:**
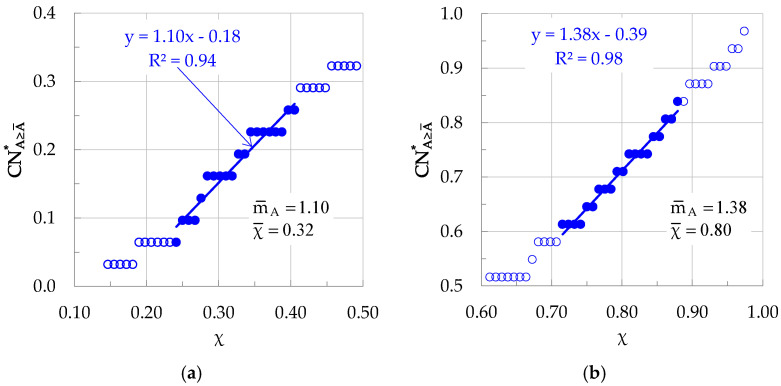
The procedure adopted for the determination of the slope ${\bar{m}}_{A}$ of the evolution of $CN_{A\geq \bar{A}}^{*}$ in the natural time domain. (**a**) Relatively small χ-values; (**b**) relatively high χ-values. The filled circular symbols indicate the k elements of the time series comprising the specific group.

Adopting now the “sliding window” technique, it is possible to determine the evolution of the slope ${\bar{m}}_{A}$ of the function $CN_{A\geq \bar{A}}^{*}$ = f(χ). In this direction, one starts by isolating a group including the first k successive elements of the $CN_{A\geq \bar{A}}^{*}$ time series. The exact value of k depends on the overall number of elements of the time series studied, being in fact a small portion of it. For the experiment analyzed here, it was set as k = 20. For this first group of elements, a linear trendline is determined and a first value of the slope ${\bar{m}}_{A}$ is calculated. “Sliding” now the first window by an arbitrary step Δk, a second group is formed. In this study, the “sliding” step was set as equal to Δk = 1. Calculating the slope of the linear trendline of this second group of elements, a second value is determined for ${\bar{m}}_{A}$, and so on. The above procedure for two groups of elements (one for relatively small values of χ and one for relatively high ones) is shown indicatively in Figure 11.

**Figure 12:**
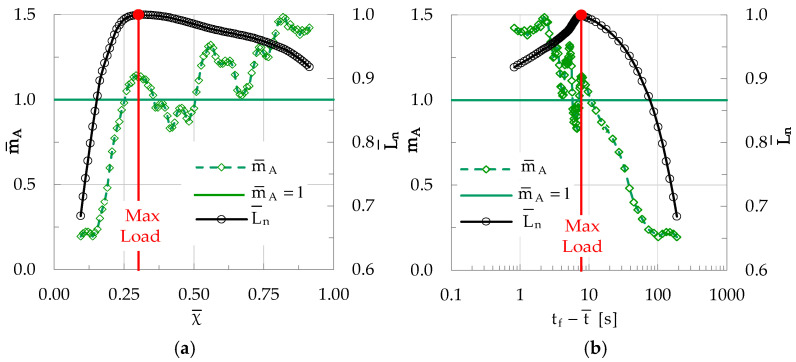
The evolution of ${\bar{m}}_{A}$ in (**a**) the natural time domain (i.e., versus $\bar{\chi }$) and (**b**) in the conventional time domain (i.e., versus t_f_-$\bar{t}$), for a plain concrete specimen. In both figures the respective evolution of the normalized average load ${\bar{L}}_{n}$ is also plotted. The green horizontal line indicates the characteristic value ${\bar{m}}_{A}$ = 1.

Each value of ${\bar{m}}_{A}$ is then paired to the average value $\bar{\chi }$ of the natural time instants χ_i_ of the specific group of elements, rendering it possible to plot the ${\bar{m}}_{A}\;$=$\;{\bar{m}}_{A}(\chi )$ function. For the experiment described here, the respective plot is shown in Figure 12a, in which the evolution of ${\bar{m}}_{A}$ is plotted versus $\bar{\chi }$. In the same figure, the evolution of the normalized average load ${\bar{L}}_{n}$ (namely, the mean value of the loads corresponding to the instants at which each element of the specific group was recorded) is also plotted, versus $\bar{\chi }$. For comparison reasons the evolution of the same quantities (i.e., ${\bar{m}}_{A}$ and ${\bar{L}}_{n}$) is plotted in Figure 12b in the conventional time domain, i.e., versus the average time-to-failure parameter t_f_-$\bar{t}$, where $\bar{t}$ is the mean value of the time instants at which the k elements of the group under study were recorded (adopting a logarithmic scale).

It is clearly seen from Figure 12a that during the early loading stages (i.e., while the load increases towards its peak value), the slope ${\bar{m}}_{A}$ of the evolution of $CN_{A\geq \bar{A}}^{*}$ is systematically smaller than unity, suggesting that the acoustic signals generated correspond to events with an amplitude smaller than $\bar{A}$. During this stage, ${\bar{m}}_{A}$ increases relatively smoothly, attaining for the first time the value ${\bar{m}}_{A}\;$= 1 at an instant $\bar{\chi }$ ≈ 0.25, slightly before the instant at which the load is maximized (i.e., the instant with $\bar{\chi }\;$≈ 0.30). Translating this information in terms of conventional time, it is concluded that ${\bar{m}}_{A}$ reaches for the first time the limit ${\bar{m}}_{A}\;$= 1 about 3.2 s before the load attains its peak value. From this instant on, ${\bar{m}}_{A}$ keeps increasing until the maximization of the load applied. Then, as the load starts decreasing ${\bar{m}}_{A}$ decreases also, attaining values even below the ${\bar{m}}_{A}\;$= 1 limit. However, the values of ${\bar{m}}_{A}$ “recover” soon, exceeding again the limit ${\bar{m}}_{A}\;$= 1, and they keep increasing with strong fluctuations. At the instant of macroscopic fracture, the terminal value of ${\bar{m}}_{A}$ is equal to about ${\bar{m}}_{A}\;$≈ 1.5.

It is recalled at this point that the characteristic value ${\bar{m}}_{A}\;$= 1 corresponds to the constant slope of the CN* = f(χ) function, i.e., of the function of the normalized cumulative number of acoustic events plotted in terms of natural time χ (see Figure 10). An indication is thus highlighted (which will be verified in further sections) that fulfilling the condition ${\bar{m}}_{A}\;$= 1 corresponds to the production of acoustic events with increased energy content, which is a characteristic feature of the stage of rapid development of networks of microcracks, which will lead, in turn, to the stage of their coalescence and the generation of the fatal macrocrack.

### 3.2. The Evolution of the Parameter ${\bar{m}}_{A}$ for Specimens Made of Fiber-Reiforced Concrete

**Figure 13:**
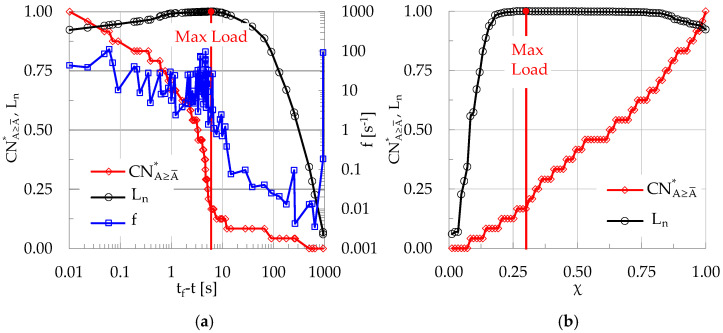
The evolution of $CN_{A\geq \bar{A}}^{*}$ (**a**) in the conventional time domain (i.e., versus the time-to-failure, t_f_-t), in juxtaposition to the instantaneous frequency of generation of acoustic events, f, and (**b**) in the natural time domain (namely, versus the dimensionless parameter χ). The respective evolution of the applied normalized load is also shown in both figures.

A characteristic experiment of the CM class of specimens (i.e., the specimens made of concrete reinforced with short metallic fibers) will be considered first in this section. During this experiment, n = 24 events with $A\geq \bar{A}$ were recorded, corresponding to about 29% of the overall number of the N = 83 events recorded. The evolution of the normalized quantity $CN_{A\geq \bar{A}}^{*}$ is plotted in Figure 13a in the conventional time domain (i.e., versus the time-to-failure parameter), and in Figure 13b in the natural time one (i.e., versus χ). In both figures the respective evolution of the normalized load, L_n_, is also plotted. The instantaneous rate of generation of acoustic events, f, is also plotted in Figure 13a. It is easily seen from Figure 13a that while $CN_{A\geq \bar{A}}^{*}$ increases very smoothly for the major portion of the loading process, it exhibits a sudden increasing trend as the load approaches its maximum value. This sudden increase is almost simultaneous with a clearly distinguishable change in the evolution of the instantaneous frequency, f, of the generation of acoustic events.

**Figure 14:**
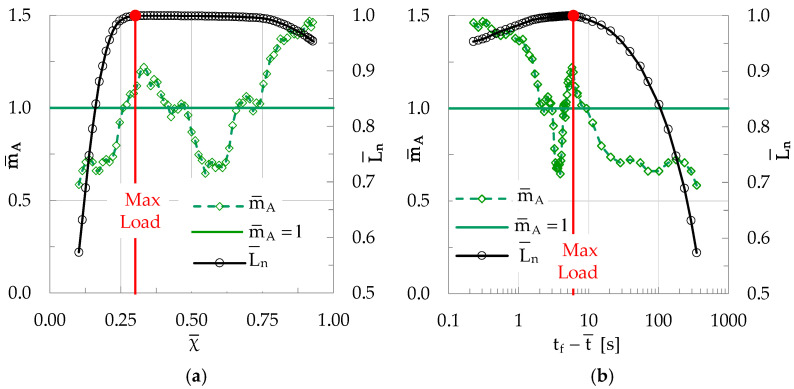
The evolution of ${\bar{m}}_{A}$ in (**a**) the natural time domain (i.e., versus $\bar{\chi }$) and (**b**) in the conventional time domain (i.e., versus t_f_-$\bar{t}$) for a specimen made of concrete reinforced with short metallic fibers (CM class). In both figures the respective evolution of the normalized average load ${\bar{L}}_{n}$ is also plotted. The green horizontal line indicates the characteristic value ${\bar{m}}_{A}$= 1.

In order to proceed with the analysis of the data of the specific experiment, groups of acoustic events were now considered, each one of them including k = 15 elements. Concerning the “sliding” step, it was again set equal to δk = 1. Following the “sliding window” procedure, described analytically in the previous section, the average slope ${\bar{m}}_{A}$ of the evolution of $CN_{A\geq \bar{A}}^{*}$ is, again, determined. The results of the above procedure are plotted in Figure 14a in the natural time domain and in Figure 14b in the conventional time one (in juxtaposition to the respective evolution of the normalized average load, ${\bar{L}}_{n}$, in both domains, i.e., conventional and natural). As deduced from Figure 14, the values of the parameter ${\bar{m}}_{A}$ are, as previously, smaller than unity during the early loading stage, exhibiting a systematic increasing tendency as the applied load is smaller than its peak value.

As the load tends to be maximized, ${\bar{m}}_{A}$ attains for the first time the limiting value ${\bar{m}}_{A}$ = 1, at a natural time instant χ ≈ 0.26, corresponding to t_f_-$\bar{t}\;$≈ 9.3 s, i.e., 9.3 s before the instant of the fracture (“conventional” for the specific class of specimens). After its maximization, the load remains almost constant for a very short time interval, during which ${\bar{m}}_{A}$ decreases significantly (well below the ${\bar{m}}_{A}\;$= 1 limit), suggesting that there is now an increased generation of events with $A\;<\bar{\;A}$. The values of ${\bar{m}}_{A}$ start increasing again only while the load starts decreasing, and they attain a terminal value equal to about ${\bar{m}}_{A}\;$= 1.5 at the “conventional” fracture instant.

**Figure 15:**
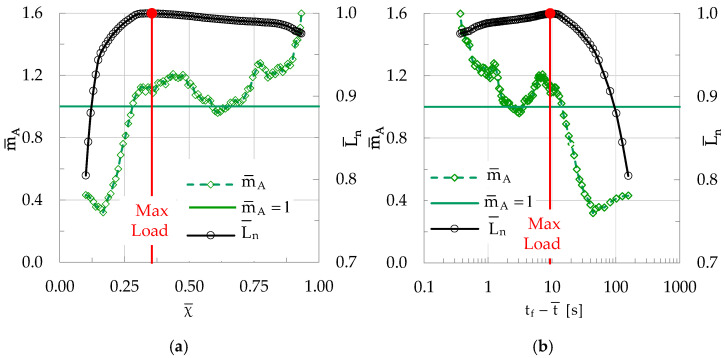
The evolution of ${\bar{m}}_{A}$ in (**a**) the natural time domain (i.e., versus $\bar{\chi }$) and (**b**) in the conventional time domain (i.e., versus t_f_-$\bar{t}$) for a specimen made of concrete reinforced with short polyolefin fibers (CP/F class). In both figures the respective evolution of the normalized average load ${\bar{L}}_{n}$ is also plotted. The green horizontal line indicates the characteristic value ${\bar{m}}_{A}\;$= 1.

As a next step, the evolution of the ${\bar{m}}_{A}$ parameter is studied for a specimen made of concrete reinforced with short polyolefin fibers, i.e., of the CP/F class. During the typical experiment analyzed here, N = 104 acoustic events were recorded, among which n = 33 (i.e., about 32% of N) fulfilled the condition $A\;\geq \bar{\;A}$. Following the “sliding window” procedure with groups including k = 20 successive events and a “sliding step” equal to δk = 1, the evolution of ${\bar{m}}_{A}$ was determined both in the natural time domain and also in the conventional time one. For the sake of brevity, the intermediate steps are here omitted, since they are identical to the ones followed for the analysis of the CM class specimen. The results of the analysis are shown in Figure 15a versus $\bar{\chi }$ (i.e., in the natural time domain), and in Figure 15b versus t_f_-$\bar{t}$ (i.e., in the conventional time domain).

As can be straightforwardly seen from Figure 15, the evolution of ${\bar{m}}_{A}$ is qualitatively similar to that of the CM specimens. Indeed, the values of ${\bar{m}}_{A}$ are systematically lower than unity during the stage at which the applied stress increases. In this stage, which covers the overwhelmingly larger portion of the test’s duration, the ${\bar{m}}_{A}$ values increase systematically. A little before the load attains its peak value, ${\bar{m}}_{A}$ attains the limiting value ${\bar{m}}_{A}$= 1 for the first time. This happens at a natural time instant χ ≈ 0.28, which corresponds to t_f_-$\bar{t}\;$≈ 14 s, i.e., 14 s before the “conventional” fracture instant. For the specific specimen, the load after its maximization starts decreasing very smoothly while ${\bar{m}}_{A}$ increases slightly beyond the ${\bar{m}}_{A}\;$= 1 limit, and then it decreases at levels around unity. As the load starts decreasing rapidly (towards its conventional fracture value), ${\bar{m}}_{A}$ increases rapidly, attaining a value ${\bar{m}}_{A}$ = 1.6 at the instant of conventional fracture.

As a last example, a specimen of the CP/P class (i.e., made of concrete reinforced with short polypropylene fibers) is considered. For the specific specimen, N = 92 acoustic events were recorded in total.

**Figure 16:**
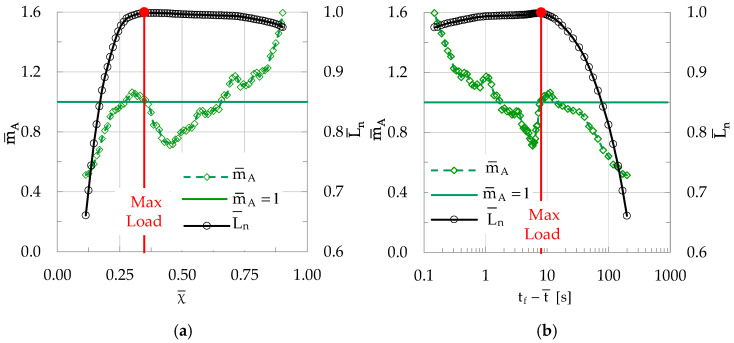
The evolution of ${\bar{m}}_{A}$ in (**a**) the natural time domain (i.e., versus $\bar{\chi }$) and (**b**) in the conventional time domain (i.e., versus t_f_-$\bar{t}$) for a specimen made of concrete reinforced with short polypropylene fibers (CP/P class). In both figures the respective evolution of the normalized average load ${\bar{L}}_{n}$ is also plotted. The horizontal green line indicates the characteristic value ${\bar{m}}_{A}\;$= 1.

The amplitude of n = 26 of them (about 28% of N) exceeded the average amplitude of the N events. As conducted for the previously discussed specimens (either made of plain or fiber-reinforced concrete), the evolution of ${\bar{m}}_{A}$ is studied both in the natural and in the conventional time domains, as exhibited in Figure 16a and Figure 16b, respectively.

It is concluded from these figures that the evolution of ${\bar{m}}_{A}$ is similar to that of both the CM and the CP/F classes. Again, ${\bar{m}}_{A}$ is systematically lower than the limiting value of one during the stage of increasing load, increasing monotonically with increasing load. At a time of 13.3 s before the instant of conventional fracture, ${\bar{m}}_{A}$ attains the limiting value of one. This instant corresponds to a natural time instant equal to χ ≈ 0.27. For the characteristic specimen discussed here, after attaining its peak value the load remains almost constant for a few seconds. During this interval ${\bar{m}}_{A}$ decreases smoothly, attaining values well below the ${\bar{m}}_{A}\;$= 1 limit. Then, as the load starts decreasing towards the conventional fracture load, ${\bar{m}}_{A}$ starts increasing rapidly, exceeding the ${\bar{m}}_{A}$ = 1 limit and reaching a value equal to about ${\bar{m}}_{A}\;$= 1.6 at the instant of conventional fracture.

## 4. Discussion

The most intriguing observation from the analysis regarding the evolution of the average slope ${\bar{m}}_{A}$ of the cumulative number of acoustic events, the amplitude of which exceeds the average of the amplitudes of all the events recorded during a specific experiment, is that it exhibits some common qualitative and quantitative characteristics independently of the composition of the material of the specimens. Namely, these common features are exhibited both for the specimens that were made of plain concrete, and also for the specimens made of concrete reinforced with all three types of short reinforcing fibers. Indeed, during the early loading stage (namely, as the level of the externally applied load is below its peak value) ${\bar{m}}_{A}$ is well below unity, suggesting a strongly pronounced rarity of acoustic events with amplitude exceeding $\bar{A}$ during this period. In this stage, the events with $A\;<\bar{\;A}$ dominate; however, ${\bar{m}}_{A}$ increases steadily for all the specimens tested. As the load increases towards its maximum value, ${\bar{m}}_{A}$ attains (for the first time) the limiting value ${\bar{m}}_{A}$ = 1 some seconds before the maximization of the load, indicating that the events with $A\;\geq \bar{\;A}$ and those with $A\;<\bar{\;A}$ reach a balance concerning their rates of production.

After attaining the value ${\bar{m}}_{A}\;$= 1, a clearly distinguishable (though temporary) decay of ${\bar{m}}_{A}$ is observed towards values even below unity as the applied load attains its peak level. This is a sign that immediately after the maximization of the load the generation of acoustic events with $A\;\geq \;\bar{A}$ becomes weaker since the energy content of the loaded element is temporarily relieved (due to the generation of the main macrocrack front which absorbs a portion of the elastic strain energy). Finally, as the load starts decreasing (either smoothly or abruptly) towards its fracture value, the values of ${\bar{m}}_{A}$ start increasing (with some secondary fluctuations), attaining values in the 1.5–1.6 interval at the instant of fracture (either actual or conventional) of the specimen.

Denoting now by ${\bar{t}}_{1}$ the average time instant at which ${\bar{m}}_{A}$ becomes equal to unity for the first time, the differences ${t_{L_{max}}-\bar{t}}_{1}$ (namely the time interval between the load maximization and the instant at which the condition ${\bar{m}}_{A}\;$= 1 is fulfilled for the first time) are recapitulated in Table 3. In the same table, the differences $t_{f}-{\bar{t}}_{1}$ (i.e., the time interval between the instant of fracture—either conventional or actual—and the instant at which the condition ${\bar{m}}_{A}\;$= 1 is for the first time fulfilled) are also included for convenience and comparison reasons.

**Figure 17:**
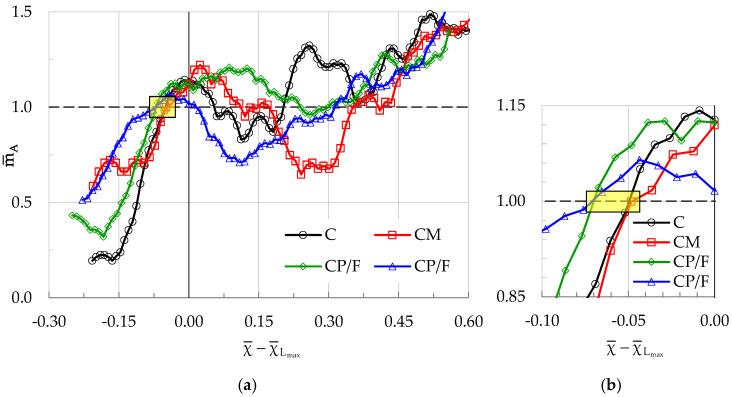
(**a**) The evolution of ${\bar{m}}_{A}$ in the natural time domain for all four classes of specimens tested; (**b**) Magnified view of the area of interest.

The common qualitative and quantitative characteristics of the evolution of ${\bar{m}}_{A}$ for the four classes of specimens tested are vividly seen in Figure 17a, in which ${\bar{m}}_{A}$ is plotted in the natural time domain in terms of the parameter $\bar{\chi }\text{-}{\bar{\chi }}_{L_{\max}}$ (i.e., assigning to the instant of load maximization the zero value of the abscissas), so that a common plot of all experiments can be visualized. The most interesting conclusion drawn from Figure 17 is that the value ${\bar{m}}_{A}\;$= 1 is attained for all four experiments in an extremely narrow interval of χ-values (see the small yellow rectangle in Figure 17a). This is more clearly seen in the detailed view of this plot, which is shown in Figure 17b.

The issue that remains to be explored further at this point is the role of the threshold set for the distinction between events with high and low amplitudes. Indeed, up to now this threshold was assumed (somehow arbitrarily) to be the average value $\bar{A}$ of the amplitudes of all the acoustic events recorded during a given experiment. The question is whether a different value for this threshold would significantly influence the conclusions that were drawn.

In this context, an experiment of the C class (specimens made of plain concrete) is here analyzed further, assigning different values to the threshold between events with low and high amplitude. For the specific experiment, the average value of the amplitudes of all the events recorded was equal to $\bar{A\;}$= 156 mV. Thus, two additional thresholds were adopted, equal to 100 mV and 300 mV, i.e., one higher and one lower than $\bar{A}$. The portion of events with amplitude exceeding the lower threshold (i.e., A ≥ 100 mV) was equal to 43%. The respective portion for the higher threshold (i.e., events with A ≥ 300 mV) was equal to 19%. Finally, for $A\;\geq \;\bar{A\;}=\;156\;\mathrm{mV}$ the respective portion of events was 31%.

**Figure 18:**
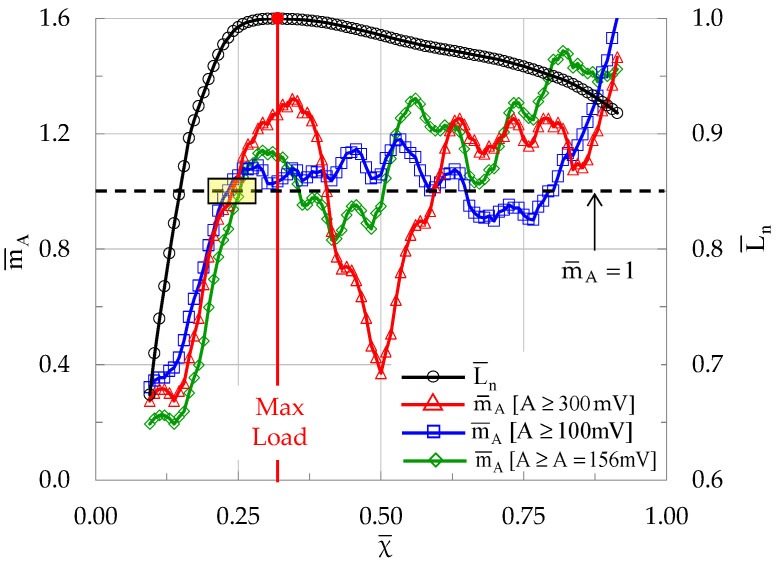
The evolution of ${\bar{m}}_{A}$ in the natural time domain (i.e., versus $\bar{\chi }$) for three different thresholds for a specimen made of plain concrete. The evolution of the normalized average load ${\bar{L}}_{n}$ is also plotted.

For these thresholds, the evolution of ${\bar{m}}_{A}$ in terms of natural time is plotted in Figure 18. It can be clearly observed from Figure 18 that the main feature of the function ${\bar{m}}_{A}\;$=$\;{\bar{m}}_{A}(\chi )$, i.e., to attain the critical limit ${\bar{m}}_{A}\;$= 1 slightly before the maximization of the externally applied load, appears for all three plots. It is quite striking that the average natural time instants at which the condition ${\bar{m}}_{A}\;$= 1 is fulfilled are very close to each other, in spite of the huge differences between the three thresholds. In fact, for the lower threshold (i.e., A ≥ 100 mV) it holds that χ_cr_ ≈ 0.22, for the highest one (i.e., A ≥ 300 mV) it holds that χ_cr_ ≈ 0.24, and for the threshold $\bar{A\;}=\;156\;\mathrm{mV}$ it holds that χ_cr_ ≈ 0.22 (see the small yellow rectangle in Figure 18). The main qualitative difference between the three plots is the level of fluctuations which increase with increasing threshold.

Before concluding this section, it is interesting to consider the outcomes concerning the temporal evolution of ${\bar{m}}_{A}$ against the respective evolution of alternative quantities that are assumed to provide pre-failure indicators, i.e., signals warning about entrance of the loaded system (or specimen) to the critical stage of impending fracture. 

**Figure 19:**
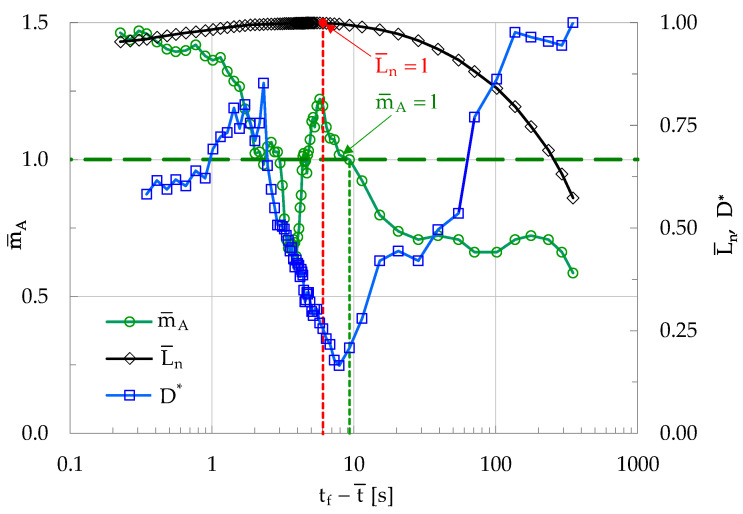
The evolution of the average normalized (over its maximum value) Euclidean distance, D*, between the sources of the acoustic events, versus the logarithm of the average time-to-failure, t_f_-$\bar{t}$, for a specimen of the CP/P class. The respective evolution of the slope ${\bar{m}}_{A}$ and that of the average normalized load ${\bar{L}}_{n}$, are also plotted.

In this context, besides the instantaneous rate, f, of the generation of acoustic events (discussed analytically in Section 3.1), the evolution of the average Euclidean distance, D, between the sources of acoustic events will be explored comparatively to that of ${\bar{m}}_{A}$. The minimization of the distance between the acoustic sources has been recently proposed as a potential pre-failure indicator [46]. In this direction, and as a characteristic example, the evolution of D for a specimen of the CP/P class is explored. For this example, D is normalized over its maximum value and is denoted as D*. The evolution of D* versus the logarithm of the average time-to-failure, t_f_-$\bar{t}$, is plotted in Figure 19 in juxtaposition to the respective evolution of the slope ${\bar{m}}_{A}$ and that of the normalized average load ${\bar{L}}_{n}$. It can be clearly seen from Figure 19 that the instant at which ${\bar{m}}_{A}$ fulfills for the first time the condition ${\bar{m}}_{A}$=1 is almost identical to the instant at which the average Euclidean distance, D*, between the acoustic sources tends to be minimized. 

It is here recalled that the minimization of this distance suggests the intense local concentration of microcracks, being, thus, a precursor of the onset of coalescence of the microcracks towards the direction of forming the fatal macrocrack front. Therefore, the minimization of D is considered as a valuable index signaling entrance of a loaded system to critical stages. In this context, and taking into account Figure 19, it can be suggested that an intrinsic interrelation exists between the mechanisms leading to the minimization of D and those forcing ${\bar{m}}_{A}$ to attain the limiting value ${\bar{m}}_{A}$=1.

**Figure 20:**
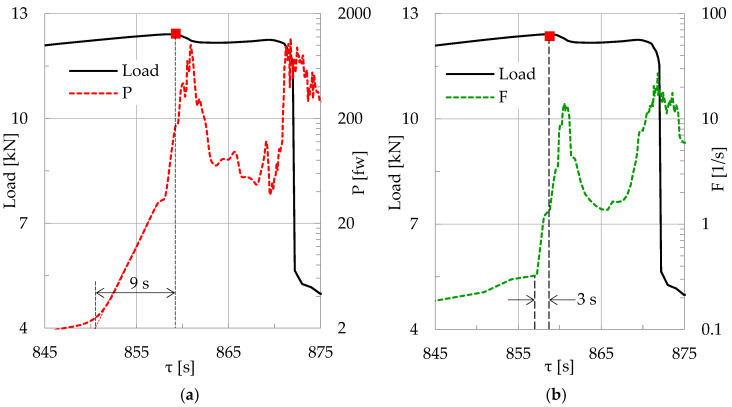
The evolution of (**a**) the power and (**b**) the F-function of the acoustic emissions versus the average time τ for a specimen made of concrete reinforced with short polyolefin fibers (CP/F class) during the very last seconds before fracture. In both figures the evolution of the respective applied load is also plotted (red squares indicate the maximum load).

In an attempt for a preliminary quantitative comparison between the outcomes of the present study (concerning the instant at which the pre-failure index is detected) versus the respective instant of traditional approaches, the data for a characteristic specimen made of concrete reinforced with short polyolefin fibers are here analyzed further. The analysis is implemented, again, according to the “sliding window” procedure, in terms of the energy of the acoustic emissions per unit time (i.e., the power P of the emissions recorded) and the average rate of generation of acoustic events, as quantified by the F-function [47]. The comparison is implemented in terms of the time interval, Δτ, between the onset of rapid increase in either F or P and the instant of maximization of the externally applied load. The results of this analysis are plotted in Figure 20, in which the power of the acoustic emissions (Figure 20a) and the F-function (Figure 20b) are plotted versus the average time, τ, focusing attention on the very last seconds before fracture. In both Figure 20a and Figure 20b the respective load applied is also plotted.

It can be seen from this figure that Δτ is equal to about Δτ ≈ 9 s for the power of the AEs and about Δτ ≈ 3 s for the F-function. Considering Figure 15, it can be seen that ${\bar{m}}_{A}$ attains its limiting value ${\bar{m}}_{A}$ = 1 about Δτ ≈ 6 s before the maximization of the load, although the onset of its rapid increase starts about Δτ ≈ 34 s before the load attains its peak value. In light of the above discussion, it is believed that ${\bar{m}}_{A}$ is indeed an interesting and potentially useful parameter from the practical engineering point of view. However, it is suggested to avoid drawing definite conclusions about its efficiency in comparison to traditional parameters before additional experimental protocols with a wider variety of materials and loading schemes are available.

## 5. Conclusions

In this study an alternative approach for the detection of early warning signals designating entrance of a loaded specimen into the stage of impending macroscopic fracture was introduced. This approach is based on the study of specific characteristics of a rather disregarded parameter of the Acoustic Emissions technique, namely, the cumulative number of acoustic events which are recorded from the onset of the loading procedure up to the fracture of the specimens.

The objective of the study was achieved by analyzing data from an experimental protocol, previously published by the authors’ team [40,41]. The protocol involved beam-shaped specimens (mechanically pre-notched at their mid-span), made of either plain or fiber-reinforced concrete, under a three-point bending loading scheme.

Along this direction, the average slope ${\bar{m}}_{A}$ of the evolution of the normalized cumulative number of acoustic events with amplitude exceeding the respective average value was analyzed both in the conventional and also in the natural time domains. The specific quantity was selected, among others, due to the fact that it is independent of the rate of generation of events depending only on the rate of generation of events with high amplitude. The analysis of the response of all four classes of specimens tested was qualitatively identical. Slightly before the applied load attains its peak value, ${\bar{m}}_{A}$ attains for the first time a limiting value equal to ${\bar{m}}_{A}$=1. The analysis of ${\bar{m}}_{A}$ in terms of natural time was once again proven advantageous since it offered a smooth distribution, highlighting details that could remain hidden if the analysis was implemented in the conventional time domain.

It could be anticipated that the quantity ${\bar{m}}_{A}$ can only be calculated after the termination of the experiment, or, equivalently, after the fracture of the specimen, because it is only after recording all the acoustic events that the average value $\bar{A}$ can be calculated. Therefore, the potentiality of ${\bar{m}}_{A}$ to provide signs considered as pre-failure indices becomes questionable. In this context, additional thresholds were considered for the amplitude of the acoustic events. It was concluded that, within specific limits, the response of ${\bar{m}}_{A}$ exhibits qualitatively similar characteristics independently of the threshold set. Along these lines, the authors’ team studies the potentialities of “instantaneous” thresholds (like, for example, moving or rolling averages). The preliminary results are very encouraging. However, and in spite of these encouraging results, it is suggested that at this stage of the research project, the approach proposed here for detecting pre-failure warning indices should be considered as supporting already existing approaches, rather than as replacing them.

Before concluding, it is worth mentioning that some inherent correlation between the evolution of ${\bar{m}}_{A}$ and that of other quantities provided by the analysis of acoustic emissions data (like, for example, the instantaneous frequency of generation of acoustic events and the average Euclidean distance between the sources of the acoustic events) was highlighted. In this context, the above-described systematic motif that was observed while analyzing the evolution of the parameter ${\bar{m}}_{A}$ in the natural time domain could provide an interesting tool (at least) for assessing the reliability of the outcomes of various approaches used to detect pre-failure indicators by taking advantage of experimental data recorded with the aid of the Acoustic Emissions technique. In any case, the above interrelations and the conclusions drawn must be further explored for a wider range of materials and loading protocols.

## Figures and Tables

**Table 1:** The composition of the concrete used for the preparation of the specimens.

Ingredient	Amount Per m^3^ of Concrete [kg]	Percentage
Coarse aggregates (16–28 mm)	669	28.0
Fine aggregates (4–16 mm)	248	10.4
Sand	987	41.3
Cement	320	13.4
Water	157	6.6
Superplasticizer	3	0.13
Reinforcing fibers	4	0.17

**Table 2:** Synopsis of the characteristics of the reinforcing fibers and the numerical values of some characteristic mechanical and acoustic quantities.

Class	Characteristics of the Reinforcing Fibers				Experimental Data					
	Type/ Content ^(^*^)^	L ^(^**^)^ [mm]	D ^(^***^)^ [mm]	Tensile Strength [MPa]	L_max_ [kN]	t_f_-t_Lmax_ [s]	N	$\bar{A}$ [mV]	n	n/N %
C	- - -	-	-	-	12.6	7.7	116	154	31	27
CM	Steel	50	1.30	690	14.6	6.1	83	147	24	29
CP/F	Polyolefin	60	0.84	465	14.2	9.3	104	155	33	32
CP/P	Polypropylene	50	0.75	625	13.8	8.0	92	114	26	28

^(^*^)^ The content of fibers was equal to 4 kg per cubic meter of concrete for all three classes of reinforced concrete specimens, ^(^**^)^ L: length of the fibers, ^(^***^)^ D: diameter of the fibers.

**Table 3:** Synopsis of the differences between the instant ${\bar{t}}_{1}$ at which ${\bar{m}}_{A}$ becomes equal to one for the first time, and the instants of load maximization ${\bar{t}}_{L_{\max}}$ and fracture t_f_.

Class	${t_{L_{max}}-\bar{t}}_{1}$ [s]	$t_{f}\text{-}{\bar{t}}_{1}$ [s]
C	3.2	10.9
CM	3.3	9.3
CP/F	4.7	14.0
CP/P	7.0	15.4

## Data Availability

The original contributions presented in this study are included in the article. Further inquiries can be directed to the corresponding author.

## References

[1] (2022). Standard Guide for Acoustic Emission Examination of Concrete Structures.

[2] RILEM Technical Committee (Masayasu Ohtsu) (2010). Recommendation of RILEM TC 212-ACD: Acoustic emission and related NDE techniques for crack detection and damage evaluation in concrete—Test method for damage qualification of reinforced concrete beams by acoustic emission. Mater. Struct..

[3] Grosse C.U., Grosse C.U., Ohtsu M., Aggelis D.G., Shiotani T. (2022). Introduction. Acoustic Emission Testing.

[4] Gholizadeh S., Leman Z., Baharudin B.T.H.T. (2015). A review of the application of acoustic emission technique in engineering. Struct. Eng. Mech..

[5] Arash Behnia A., Chai H.K., Shiotani T. (2014). Advanced structural health monitoring of concrete structures with the aid of acoustic emission. Constr. Build. Mater..

[6] Rasheed M.A., Prakash S.S., Raju G., Kawasaki Y. (2018). Fracture studies on synthetic fiber reinforced cellular concrete using acoustic emission technique. Constr. Build. Mater..

[7] Lacidogna G., Piana G., Accornero F., Carpinteri A. (2020). Multi-technique damage monitoring of concrete beams: Acoustic emission, digital image correlation, dynamic identification. Constr. Build. Mater..

[8] Triantis D., Sarlis N.V., Loukidis A., Pasiou E.D., Stavrakas I., Kourkoulis S.K. (2023). Criticality indices provided by the evolution of pressure stimulated currents and acoustic emissions in the natural time domain. Theor. Appl. Fract. Mech..

[9] Li W., Jiang Z., Yang Z. (2017). Acoustic characterization of damage and healing of microencapsulation-based self-healing cement matrices. Cem. Concr. Compos..

[10] Monteiro P., Miller S., Horvath A. (2017). Towards sustainable concrete. Nat. Mater..

[11] Farnam Y., Geiker M.R., Bentz D., Weiss J. (2015). Acoustic emission waveform characterization of crack origin and mode in fractured and ASR damaged concrete. Cem. Concr. Compos..

[12] Su H., Hu J., Tong J., Wen Z. (2012). Rate effect on mechanical properties of hydraulic concrete flexural-tensile specimens under low loading rates using acoustic emission technique. Ultrasonics.

[13] Triantis D., Pasiou E.D., Stavrakas I., Kourkoulis S.K. (2022). Hidden affinities between electric and acoustic activities in brittle materials at near-fracture load levels. Rock Mech. Rock Eng..

[14] Yan X., Su H., Ai L., Soltangharaei V., Xu X., Yao K. (2023). Study on stage characteristics of hydraulic concrete fracture under uniaxial compression using acoustic emission. Nondestruct. Test. Eval..

[15] Triantis D., Stavrakas I., Loukidis A., Pasiou E.D., Kourkoulis S.K. (2023). A study on the fracture of cementitious materials in terms of the rate of acoustic emissions in the natural time domain. Appl. Sci..

[16] Wu K., Chen B., Yao W. (2001). Study of the influence of aggregate size distribution on mechanical properties of concrete by acoustic emission technique. Cem. Concr. Res..

[17] Triantis D., Pasiou E.D., Stavrakas I., Kourkoulis S.K. (2024). Revealing the proximity of concrete specimens to their critical damage level by exploring the cumulative counts of the acoustic emissions in the natural time domain. Materials.

[18] Vidya Sagar R., Raghu Prasad B.K. (2011). An experimental study on acoustic emission energy as a quantitative measure of size independent specific fracture energy of concrete beams. Constr. Build. Mater..

[19] Li P., Zhang W., Ye Z., Wang Y., Yang S., Wang L. (2022). Analysis of acoustic emission energy from reinforced concrete sewage pipeline under full-scale loading test. Appl. Sci..

[20] Vidya Sagar B.K., Raghu Prasad B.K., Shantha Kumar S. (2012). An experimental study on cracking evolution in concrete and cement mortar by the b-value analysis of acoustic emission technique. Cem. Concr. Res..

[21] Gu Q., Ma Q., Tan Y., Jia Z., Zhao Z., Huang D. (2019). Acoustic emission characteristics and damage model of cement mortar under uniaxial compression. Constr. Build. Mater..

[22] Iturrioz I., Lacidogna G., Carpinteri A. (2013). Acoustic emission detection in concrete specimens: Experimental analysis and lattice model simulations. Int. J. Damage Mech..

[23] Lei X. (2019). Evolution of b-value and fractal dimension of acoustic emission events during shear rupture of an immature fault in granite. Appl. Sci..

[24] Carnì D.L., Scuro C., Lamonaca F., Olivito R.S., Grimaldi D. (2017). Damage analysis of concrete structures by means of acoustic emissions technique. Compos. Part B Eng..

[25] Gutenberg B., Richter C.F. (1944). Frequency of earthquakes in California. Bull. Seismol. Soc. Am..

[26] Cox S.J.D., Meredith P.G. (1993). Microcrack formation and material softening in rock measured by monitoring acoustic emissions. Int. J. Rock Mech. Min. Sci. Geomech. Abstr..

[27] Colombo I.S., Main I.G., Ford M.C. (2003). Assessing damage of reinforced concrete beam using b-value analysis of acoustic emission signal. J. Mater. Civ. Eng..

[28] Rao M.V.M.S., Prasanna Lakshmi K.J. (2005). Analysis of b-value and improved b-value of acoustic emissions and accompanying rock fracture. Curr. Sci..

[29] Aggelis D.G., Soulioti D.V., Sapouridis N., Barkoula N.M., Paipetis A.S., Matikas T.E. (2011). Acoustic emission characterization of the fracture process in fibre reinforced concrete. Constr. Build. Mater..

[30] Loukidis A., Stavrakas I., Triantis D. (2022). Electrical methods for sensing damage in cement mortar beams combined with acoustic emissions. Materials.

[31] Varotsos P., Sarlis N.V., Skordas E.S. (2011). Natural Time Analysis: The New View of Time. Precursory Seismic Electric Signals, Earthquakes and Other Complex Time-Series.

[32] Varotsos P.A., Sarlis N.V., Tanaka H.K., Skordas E.S. (2005). Some properties of the entropy in the natural time. Phys. Rev. E.

[33] Varotsos P., Sarlis N., Skordas E. (2023). Natural Time Analysis: The New View of Time. Advances in Disaster Prediction Using Complex Systems.

[34] Varotsos P.A., Skordas E.S., Sarlis N.V., Christopoulos S.-R.G. (2024). Review of the natural time analysis method and its applications. Mathematics.

[35] Vallianatos F., Michas G., Benson P., Sammonds P. (2013). Natural time analysis of critical phenomena: The case of acoustic emissions in triaxially deformed Etna basalt. Phys. A.

[36] Niccolini G., Manuello A., Marchis E., Carpinteri A. (2017). Signal frequency distribution and natural-time analyses from acoustic emission monitoring of an arched structure in the Castle of Racconigi. Nat. Hazards Earth Syst. Sci..

[37] Loukidis A., Pasiou E.D., Sarlis N.V., Triantis D. (2020). Fracture analysis of typical construction materials in natural time. Phys. A.

[38] Niccolini G., Potirakis S.M., Lacidogna G., Borla O. (2020). Criticality hidden in acoustic emissions and in changing electrical resistance during fracture of rocks and cement-based materials. Materials.

[39] Friedrich L.F., Cezar É.S., Colpo A.B., Tanzi B.N.R., Sobczyk M., Lacidogna G., Niccolini G., Kosteski L.E., Iturrioz I. (2022). Long-range correlations and natural time series analyses from acoustic emission signals. Appl. Sci..

[40] Triantis D., Stavrakas I., Loukidis A., Pasiou E.D., Kourkoulis S.K. (2023). Spatio-temporal distribution of the sources of acoustic events in notched fiber-reinforced concrete beams under three-point bending. Materials.

[41] Triantis D., Tsaousi D.K., Stavrakas I., Pasiou E.D., Douvis P., Kourkoulis S.K. (2020). Electric and acoustic activity in notched fiber-reinforced concrete beams under three-point bending. Mater. Today Proc..

[42] (2019). Testing Hardened Concrete—Part 2: Making and Curing Specimens for Strength Tests.

[43] Blazy J., Blazy R. (2021). Polypropylene fiber reinforced concrete and its application in creating architectural forms of public spaces. Case Stud. Constr. Mater..

[44] Alberti M.G., Enfedaque A., Gálvez J.C. (2017). Fibre reinforced concrete with a combination of polyolefin and steel-hooked fibres. Comp. Struct..

[45] Bos F.P., Bosco E., Salet T.A.M. (2019). Ductility of 3D printed concrete reinforced with short straight steel fibers. Virtual Phys. Prototyp..

[46] Triantis D., Stavrakas I., Pasiou E.D., Kourkoulis S.K. (2024). Exploring the acoustic activity in brittle materials in terms of the position of the acoustic sources and the power of the acoustic signals—Part II: Applications. Forces Mech..

[47] Triantis D., Kourkoulis S.K. (2018). An alternative approach for representing the data provided by the acoustic emission technique. Rock Mech. Rock Eng..

